# Growth of Less than 20 nm SnO Nanowires Using an Anodic Aluminum Oxide Template for Gas Sensing

**DOI:** 10.3390/mi11020153

**Published:** 2020-01-30

**Authors:** Bo-Chi Zheng, Jen-Bin Shi, Hsien-Sheng Lin, Po-Yao Hsu, Hsuan-Wei Lee, Chih-Hsien Lin, Ming-Way Lee, Ming-Cheng Kao

**Affiliations:** 1 Ph.D. Program of Electrical and Communications Engineering, Feng Chia University, Taichung 40724, Taiwan; bochi0020@gmail.com (B.-C.Z.); shemglin@ms63.hinet.net (H.-S.L.); rocklee3001g@gmail.com (H.-W.L.); 2 Department of Electrical Engineering, Feng Chia University, Taichung 40724, Taiwan; yao210131@gmail.com (P.-Y.H.); m0729970@o365.fcu.edu.tw (C.-H.L.); 3 Institute of Nanoscience and Department of Physics, National Chung Hsing University, Taichung 40227, Taiwan; mwl@phys.nchu.edu.tw; 4 Department of Electronic Engineering, Hsiuping University of Science and Technology, Taichung 41280, Taiwan; kmc@mail.hust.edu.tw

**Keywords:** anodic aluminum oxide (AAO), electrochemical deposition, nanowire, SnO

## Abstract

Stannous oxide (SnO) nanowires were synthesized by a template and catalyst-free thermal oxidation process. After annealing a Sn nanowires-embedded anodic aluminum oxide (AAO) template in air, we obtained a large amount of SnO nanowires. SnO nanowires were first prepared by electrochemical deposition and an oxidization method based on an AAO template. The preparation of SnO nanowires used aluminum sheet (purity 99.999%) and then a two-step anodization procedure to obtain a raw alumina mold. Finally, transparent alumina molds (AAO template) were obtained by reaming, soaking with phosphoric acid for 20 min, and a stripping process. We got a pore size of < 20 nm on the transparent alumina mold. In order to meet electroplating needs, we produced a platinum film on the bottom surface of the AAO template by using a sputtering method as the electrode of electroplating deposition. The structure was characterized by X-ray diffraction (XRD). High resolution transmission electron microscopy (HRTEM) and field emission scanning electron microscopy (FESEM) with X-ray energy dispersive spectrometer (EDS) were used to observe the morphology. The EDS spectrum showed that components of the materials were Sn and O. FE-SEM results showed the synthesized SnO nanowires have an approximate length of ~10–20 μm with a highly aspect ratio of > 500. SnO nanowires with a Sn/O atomic ratio of ~1:1 were observed from EDS. The crystal structure of SnO nanowires showed that all the peaks within the spectrum lead to SnO with a tetragonal structure. This study may lead to the use of the 1D structure nanowires into electronic nanodevices and/or sensors, thus leading to nano-based functional structures.

## 1. Introduction

Over the past years, there has been an enormous interest in the research and development of semiconducting nanostructures due to their potential in the application and in electronic devices as well as in the sensor field [1,2,3,4,5]. Chemical sensors based on semiconductor nanostructures are expected to have a significantly enhanced performance compared to thin films, due to their high surface-volume ratio. They are expected to be more stable and sensitive [6]. It would also reduce the response and recovery time. Consequently, semiconducting oxides such as ZnO, ln_2_O_3_ and SnO_2_ have been synthesized into many nanostructures such as one-dimensional (1D) nanowires, and they have also been combined as nanodevices and/or sensors [7,8,9]. The nanostructures can be synthesized by several methods such as PVD, laser ablation, chemical vapor deposition, electrochemical deposition, thermal evaporation and gas-deposition [10,11,12,13,14,15,16,17]. Oxides have two major characteristics: multiple valence ions and anion defects. Any change will change the proportion of electrons and holes, which will affect the properties of oxides such as light, electricity and magnetism. Therefore, the adjustment of the process parameters can produce the different materials required for various components. These oxides have been recognized as smart materials or functional materials [18]. One-dimensional (1D) metal oxide nanostructure-based sensing devices are of great importance for significantly improving the performance of sensors built using thin-film technology. Due to the excellent physical and chemical properties resulting from their reduced sizes, 1D metal oxide nanostructures like nanowires have been regarded as promising candidates for use as sensing units, and they have drawn much attention recently [19,20,21,22]. We wanted to use an anodic aluminum oxide (AAO) template for synthesizing one-dimensional nanowires because the AAO template has straight 1D porous channels [23,24]. We then used the AAO template to prepare SnO one-dimensional nanowires by electrochemical deposition.

Stannous oxide (SnO) is a tetragonal lattice p-type semiconductor with a good drift rate and high transmittance because SnO is a p-type, wide energy gap (*E*_g_~3.5 eV) semiconductor [25,26]. The crystal structure of the stannous oxide is the orthorhombic system structure. Four oxygen atoms are centered on the tin and two tin centers are centered on oxygen. It is different from general semiconductor materials. The stannous oxide nanowires have broad application potential, and the stannous oxide thin film light transmittance in the visible light region (400–800 nm) can reach 86% or more [27,28,29]. It thus has a high visible light range transmittance and it can realize low power, high-performance complementary circuits and can provide better performance in organic light-emitting diodes (OLEDs). Compared to thin-film transistors, the nanowires transistors exhibit five times higher mobility and one order of magnitude lower subthreshold swing. SnO nanowires are smaller than Cu_2_O nanowires and their threshold voltages are lower than those of SnO thin films [30]. Gas sensors based on nanowires have been demonstrated to be excellent candidates for ultrahigh sensitivity due to their high surface-to-volume ratio. A large surface-to-volume ratio means that a significant fraction of atoms (or molecules) are in a great quantity on the surface. Therefore, the reaction between target gas and reactive chemical species on the surface can occur more readily. Some previous studies have proved that template-based assembly was an easy and versatile way to obtain uniform and ordered nanoscale structured films [23,24]. Anodic alumina (AAO) template has coordinate orifices whose length is from 1 to 100 µm and diameters range from 10 to 200 nm, depending on the process conditions. Our previous studies reported the synthesis of fabricated nanometer-sized wires of various materials [31,32,33]. In this work, we describe the synthesis of a novel 20 nm SnO nanowires (p-type wide-gap semiconductor) based on AAO using electrodeposition and oxidization methods. We consider that the SnO nanowires may prove useful for gas sensors in the future.

## 2. Synthesis Procedure and Methods

In this study, we use an AAO template to electroplate Sn nanowires by electrochemical deposition. After electroplating, we put the Sn nanowires template into a high-temperature vacuum furnace for oxidation to obtain SnO nanowires.

### 2.1. Synthesizing Procedure of AAO Template

**Figure 1:**
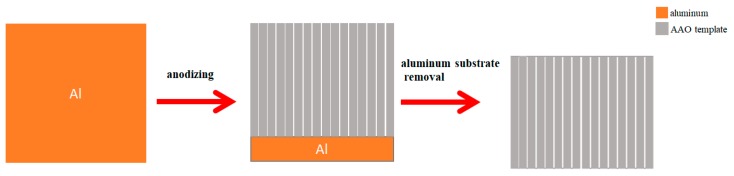
Schematic representation of the synthesizing procedure for preparing the AAO template.

First, the aluminum surface is anodized using a constant potential in a sulfuric acid electrolyte. An AAO template with a nanoporous structure is initially formed. We used a mixed solution of chromic acid and phosphoric acid to remove part of the oxide layer, followed by a second anodization under the same conditions as the first anodization. We can thus get a highly identical AAO template on the aluminum surface. The aluminum substrate was dissolved by HgCl_2_. Finally, the pore size of the AAO template was increased using phosphoric acid etching [31,32,33]. The synthesis procedure for preparing the AAO template is shown in Figure 1.

### 2.2. Synthesizing Procedure of SnO Nanowires

In this part, we used electrochemical deposition to get Sn nanowires. The AAO film is an insulator material, so we need the AAO template to have a metal electrode layer for electrochemical deposition. We used a sputtering machine to sputtering a dense and thick metal electrode layer of Pt (about 400 nm). Such a thick Pt layer is enough to provide a good conductive electrode. Furthermore, the plating liquid is an acidic liquid, so we need a layer of Pt electrode layer with good conductivity and not corroded by acidic liquids. We used stannous chloride (SnCl_2_) and boric acid (H_3_BO_3_) for the Sn nanowire electrochemical deposition. The AC and DC plating voltage cause electrochemical deposition, and we can get Sn nanowires in the AAO template. After electrochemical deposition, we put the AAO template to a high-temperature annealing furnace for annealing, then we removed the AAO template. 

**Figure 2:**
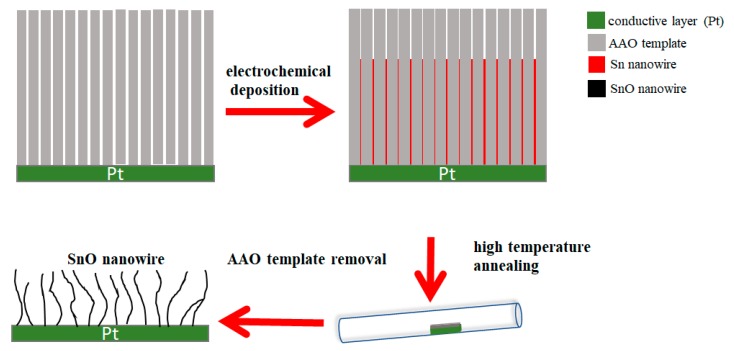
Schematic representation of the synthesizing procedure of SnO nanowires.

We can get a sample of SnO nanowires fixed on the platinum electrode layer. In order to detach the SnO nanowires from the platinum electrode layer we put the sample in alcohol and separate the Pt electrode layer using an ultrasonic oscillator. The schematic representation of the SnO nanowires synthesis procedure is shown in Figure 2. After filtering out the Pt electrode layer in alcohol, we can obtain isolated SnO nanowires.

### 2.3. A Gas-Sensor and Equipment for Sensing CO_2_

**Figure 3:**
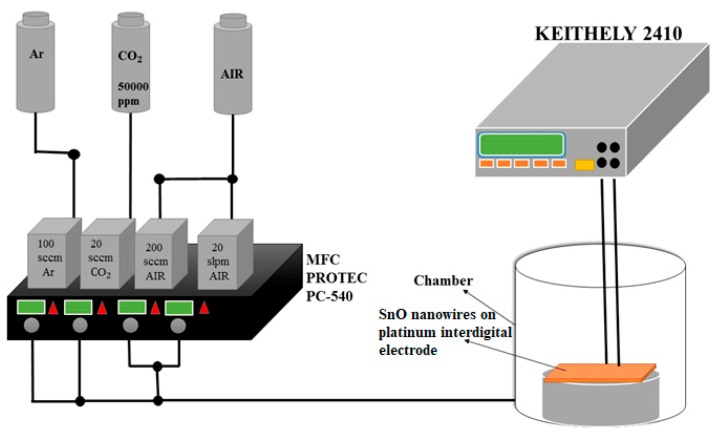
Schematic illustration of **a** gas sensor.

In this part, we dropped the obtained SnO nanowires on the platinum interdigitated electrode sensing element, put it into a vacuum cavity, and then evacuate the cavity to 1.2 × 10^−2^ torr. Figure 3 shows the equipment for sensing CO_2_. We measured the resistance of the sample with a pulsed DC power supply (2410 DC, KEITHLEY, Solon, Ohio, USA). We put CO_2_ into the vacuum chamber for 10 min. We observed the change of the resistance value and we evacuated the vacuum chamber again. After testing three times, we measured the change of the resistance value. We used a range of testing values. 

## 3. Results

About the results, we observed the AAO template by using FE-SEM. Each channel in the AAO template was clearly observed. The length of the channels and pore size were also estimated. The formation of SnO nanowires was carried out by electrochemical deposition and an oxidization method on the alumina nanoporous template prepared in the first part by sputtering a layer of platinum (Pt) on the back of the alumina template.

### 3.1. Synthesizing the AAO Template

**Figure 4:**
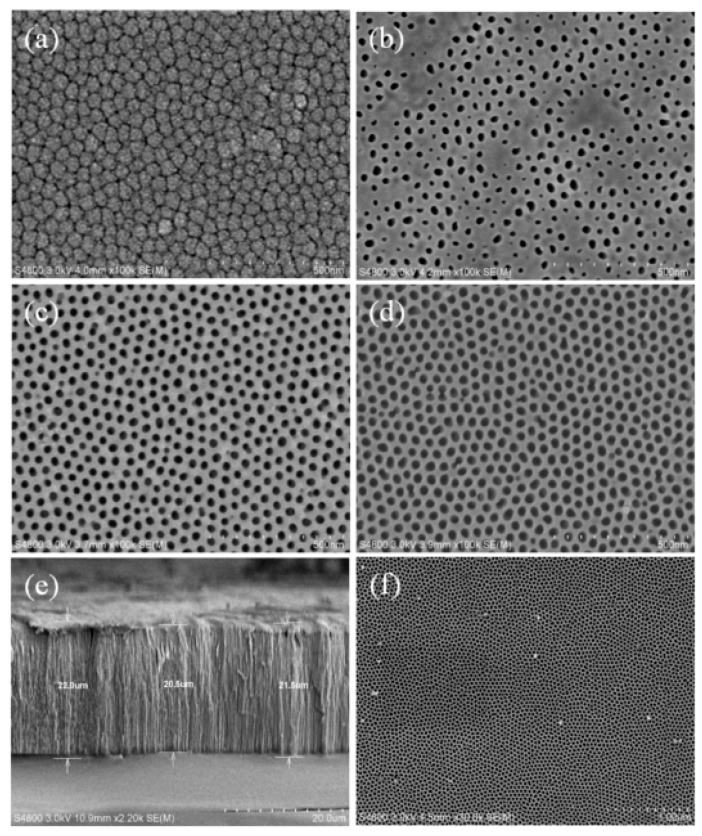
SEM images by dipping the AAO template for the different time: (**a**) 15 min, (**b**) 19 min, (**c**) 20 min, (**d**) 25 min. (**e**) The cross-section of the AAO template. (**f**) The AAO template of form pore size around 20 nm.

In the first part, the AAO template was formed in five steps. The first and second steps involved the anode treatment with 2.6 wt % sulfuric acid at 5°C and phosphoric acid with chromic acid at a temperature of 60°C for 6 h for forming the AAO template. The third and fourth steps used mercury chloride at room temperature and phosphoric acid at a temperature of 30 °C for an optimal time for completing the AAO template. In the final step, we wanted to obtain a completely through channel in the alumina template. We dipped the AAO template in phosphoric acid at a temperature of 30°C for different times. Figure 4 shows the top view of the AAO templates obtained by dipping in a phosphoric acid solution for different times. In Figure 4a, no holes are observed in the top view of the surface of the AAO template after the template was dipped in a phosphoric acid solution for 15 min. In Figure 4b, showing the AAO template after it was dipped in a phosphoric acid solution for 19 min, we observe some holes on the surface of the AAO template with a hole size around 10–20 nm. These holes had a chance to form many different channels. In Figure 4c, which shows the AAO template that was dipped in a phosphoric acid solution for 20 min, we observe many holes on the surface of the AAO template with an average size around 20–25 nm. In the result showed that the AAO template was dipped in a phosphoric acid solution for 20 min. In Figure 4d, we observe many holes on the surface of the AAO template with the average size around 30–40 nm. This result was obtained when the AAO template was dipped in a phosphoric acid solution for 25 min. According to the abovementioned descriptions, we determined the optimal time for dipping the AAO template in a phosphoric acid solution was 20 min. We used this AAO template obtained by dipping the AAO template in a phosphoric acid solution for 20 min to get thinner nanowires.

We also observed the AAO template with a tubular-like structure in the cross-section of the alumina film firstly. The cross-section of the AAO template is seen clearly in Figure 4e. Each of the channels was around 20 to 22 μm in length. We choose the AAO template which was shown to form a pore size of around 20 nm. The top view of this AAO template is shown in Figure 4f.

### 3.2. Synthesizing the SnO Nanowires

**Figure 5:**
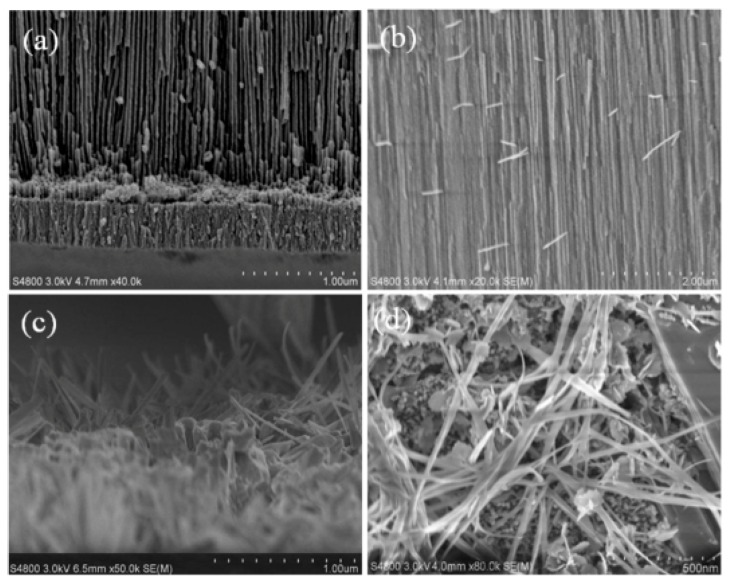
(**a**) FE-SEM image of the sectional view for sputtering a layer of platinum on the AAO template surface. (**b**) FE-SEM image of the electrodepositing the Sn material in the AAO template. (**c**) FE-SEM image of the cross-section of the SnO nanowires. (**d**) FE-SEM image of the top view of the SnO nanowires.

The formation of SnO nanowires was carried out by electrochemical deposition and an oxidization method on an alumina nanoporous template. We prepared the AAO template in the second part by sputtering a layer of platinum on the back of the alumina template as seen in Figure 5a.

In the second part of the electrochemical deposition method, we electrodeposited Sn nanowires in the AAO template by using a plating solution. The plating solution was mixed 0.01 M SnCl_2_ and 0.05 M H_3_BO_3_. The plating conditions were AC 10 V and DC 4 V. We filled the Sn material in the AAO template by using electrochemical deposition. The AAO template is also shown clearly in the cross-section in Figure 5b. We observed that the widths of each of the nanowires in Figure 5b was around 20 nm. After electrodepositing the Sn material in the AAO template, we put the AAO template in vacuum-sealed glass ampoules at 500 °C for 4 h. After annealing the AAO template, we put the AAO template in 0.3 M NaOH for 15 min. A nanowire having a diameter of < 20 nm was produced. The cross-section of the nanowires is shown clearly in Figure 5c. We observed that the widths of each of the nanowires in Figure 5c was less than 20 nm. We also observed that the length of many of the nanowires in Figure 5d was over 1 μm. 

### 3.3. Analysis of the SnO Nanowires

**Figure 6:**
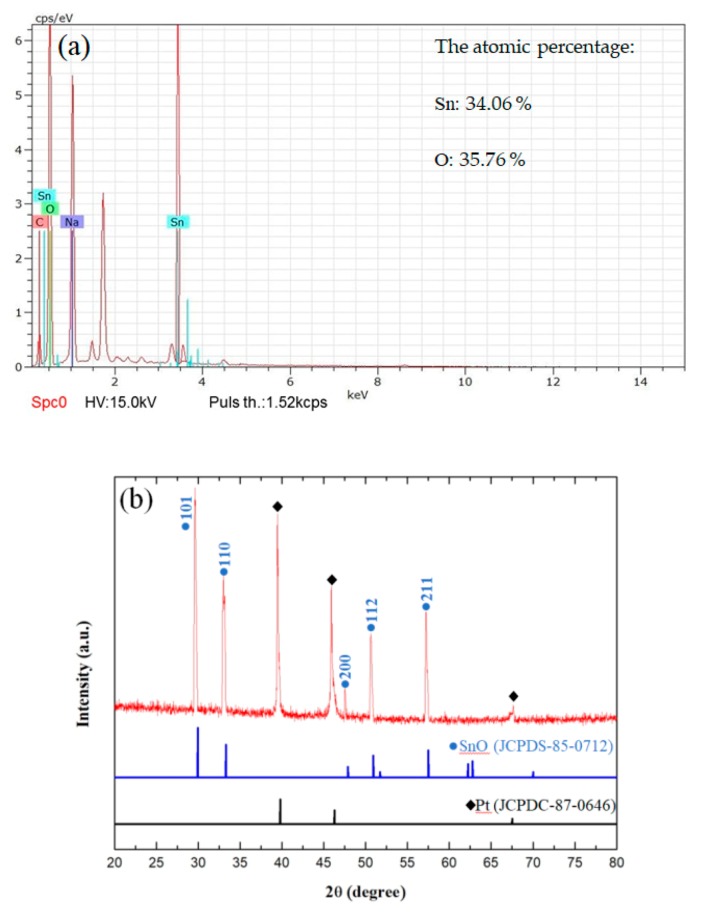

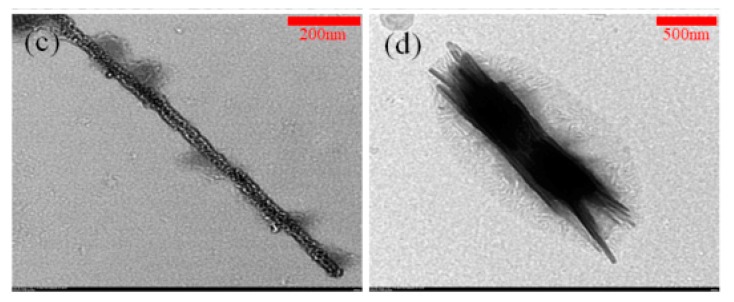
(**a**) Energy dispersive X-ray spectroscopy (EDS) of the SnO nanowires oxidized at 500 °C for 4 h; (**b**) XRD patterns of SnO nanowires on cluster oxidized at 500 °C for 4 h; (**c**) Transmission Electron Micrograph (TEM) images of single SnO nanowire and (**d**) SnO nanowires cluster.

We analyzed the SnO nanowires by using their EDS spectrum and XRD patterns. Figure 6 shows the XRD patterns and EDS spectrum of the SnO films synthesized by annealing the AAO temple with Sn at 500 °C for 4 h. 

Figure 6a shows the EDS spectrum after oxidizing the Sn nanowires. In this EDS spectrum the atomic percentages of Sn and O were shown to be 34.06% and 35.76%, respectively. The molar ratio was around 1:1. Figure 6b shows the XRD of a SnO nanowires cluster after oxidizing at 500 °C for 4 h. The SnO nanowires in Figure 6b were identical to the in situ sample (JCPDS 85-0712). We also observed the SnO nanowires by HR-TEM. A HR-TEM image of an isolated single SnO nanowire is shown in Figure 6c. A SnO nanowires cluster is shown in Figure 6d.

### 3.4. Sensing the CO_2_ by Using the SnO Nanowires

We dropped alcohol with SnO nanowires on a platinum interdigitated electrode for a testing sample. We put the sample of the interdigitated electrode sensing element in a vacuum chamber, and we measured the resistance change of the sample in a vacuum environment at room temperature [34]. We wanted to eliminate the impact of the general atmosphere. We also let the component be in direct contact with the test gas. The test gases were mixed air and carbon dioxide gas (5000 ppm). We set up the test environment to reach a pressure of 1.2 × 10^−2^ torr firstly. We used a mass flow controller (MFC, PROTEC PC-540, Junsun, Taipei, Taiwan) to release the gas flow in at a pressure of 2.1 × 10^−1^ torr. 

**Figure 7:**
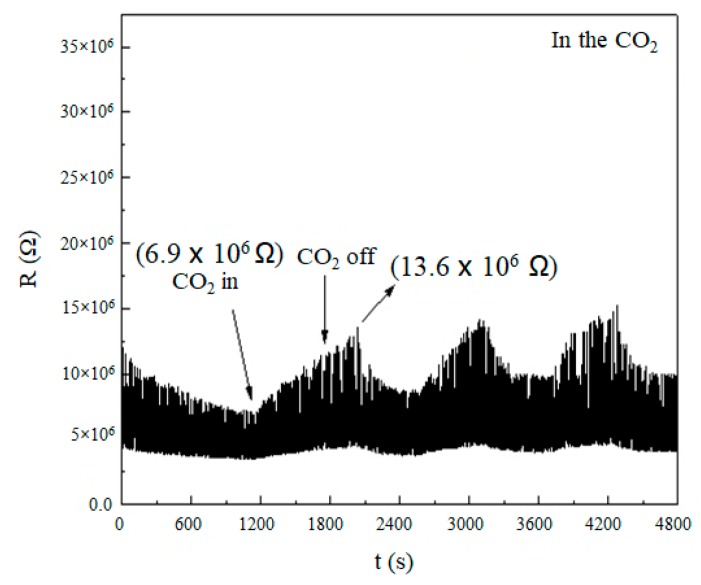
The result was for sensing the CO_2_ by using SnO nanowires on the platinum interdigitated electrode sensing element.

As shown in Figure 7, the measurement was performed using SnO nanowires which reacted with the air and carbon dioxide. We observed the test value of Figure 7 for testing the gas sensor. The change rate of resistance in CO_2_ in Figure 7 was 49%.

We show Table 1 for comparison of the CO_2_ sensing properties of our proposed device with reported work. Willa et al. reported that a simple strategy achieved an enhancement of the electrical properties required for the utilization of PILs-based CO_2_ sensors [35]. This concept can be easily extended to other electronic devices in the future. Srinives et al. reported that good sensing performance was observed upon exposing a PEI-PANI device to 50-5000 ppm CO_2_ in the presence of humidity with negligible interference from ammonia, carbon monoxide, methane and nitrogen dioxide [36]. Hafiz et al. reported an ambient CO_2_ chemiresistor platform based on a nanoporous electrically conducting two dimensional metal−organic framework (2D MOF) [37]. This CO_2_ sensor can improve upon previous materials and approaches because of its selective sensing of CO_2_ under ambient conditions, where relative humidity (RH) and other atmospheric contaminants exist. Star et al. reported that carbon nanotube field-effect transistors (NTFETs) coated with poly (ethyleneimine) (PEI) and starch polymers exhibit electrical conductance changes upon exposure to CO_2_ gas in air at ambient temperature [38]. We present SnO nanowires which are a kind of p-type semiconductor for sensing CO_2_ gas in air at ambient temperature.

## 4. Conclusions

By ujsing AC/DC to get Sn nanowires via an AAO template, AC can prevent excessive tin ions from blocking the AAO surface during electrochemical deposition to facilitate the formation of Sn nanowires. AC 10 V/DC 4 V is used to form Sn nanowires. Sn nanowires are annealed at a high temperature (500°C) to transform them into SnO nanowires. The as-obtained products were examined using diverse techniques including X-ray powder diffraction (XRD), field-emission scanning electron microscopy (FE-SEM) and high-resolution TEM. The results indicate that the SnO nanowires have a tetragonal structure. The SnO nanowire structures have a diameter of < 20 nm and lengths of 10–20 μm. These SnO nanowires could be used for electronic nanodevices and/or sensors like gas sensors in the future.

## Figures and Tables

**Table 1:** Comparison of CO_2_ sensing properties with reported work.

Materials	T (°C)	Concentration (ppm)	Sensitivity (% ppm^−1^)	Response Time (min)	Ref.
Poly- nanoparticle (ionic liquid)	Room temperature	150–2400	~0.004	35 min	[35]
PEI-functionalized PANI film	Room temperature	50–5000	~0.00714	>10 min	[36]
Reduced graphene oxide	Room temperature	0–1500	0.0118	4 min	[37]
PeI-starch-functionalized CNTs	Room temperature	0–1500	0.000101	~15 s (100%)	[38]
SnO nanowire	Room temperature	5000	0.000098	5 min	this work

## References

[1] Salah N., Al-Shawafi W.M., Habib S.S., Azam A., Alshahrie A. (2016). Growth-controlled from SnO_2_ nanoparticles to SnO nanosheets with tunable properties. Mater. Des..

[2] Lu C., Wang J., Wang A., Wang Y., Meng D., Zhu Z. (2017). Study on the structure feature of SnO micro/nanostructure with interesting distribution characteristic of concentric annulus. Mater. Lett..

[3] Rezalou S., Öznülüer T., Demir Ü. (2018). One-pot electrochemical fabrication of single-crystalline SnO nanostructures on Si and ITO substrates for catalytic, sensor and energy storage applications. Appl. Surf. Sci..

[4] Kim M.-R., Lee I., Kim K.S., Kim K.-C. (2019). Growth of free-standing SnO nanostructures on single layer graphene. Mater. Lett..

[5] Magdalane C.M., Kanimozhi K., Arularasu M., Ramalingam G., Kaviyarasu K. (2019). Self-cleaning mechanism of synthesized SnO_2_/TiO_2_ nanostructure for photocatalytic activity application for waste water treatment. Surfaces Interfaces.

[6] Amalathas A.P., Alkaisi M.M. (2019). Nanostructures for light trapping in thin film solar cells. Micromachines.

[7] Samà J., Prades J.D., Casals O., Barth S., Gracia I., Cané C., Domènech-Gil G., Hernández-Ramírez F., Romano-Rodríguez A. (2015). Low-cost fabrication of zero-power metal oxide nanowire gas sensors: Trends and challenges. Procedia Eng..

[8] Vallejos S., Grácia I., Chmela O., Figueras E., Hubálek J., Cané C. (2016). Chemoresistive micromachined gas sensors based on functionalized metal oxide nanowires: Performance and reliability. Sens. Actuators B Chem..

[9] Schipani F., Miller D.R., Ponce M.A., Aldao C.M., Akbar S.A., Morris P.A., Xu J.C. (2017). Conduction mechanisms in SnO_2_ single-nanowire gas sensors: Animpedance spectroscopy study. Sens. Actuators B Chem..

[10] Schelfhout R., Strijckmans K., Depla D. (2018). Anomalous effects in the aluminum oxide sputtering yield. J. Phys. D Appl. Phys..

[11] Sun H., Kuo T.-Y., Chen S.-C., Chen Y.-H., Lin H.-C., Pour Yazdi M.A., Billard A. (2019). Contribution of enhanced ionization to the optoelectronic properties of p-type NiO films deposited by high power impulse magnetron sputtering. J. Eur. Ceram. Soc..

[12] El Beainou R., Martin N., Potin V., Pedrosa P., Pour Yazdi M.A., Billard A. (2017). Correlation between structure and electrical resistivity of W-Cu thin films prepared by GLAD co-sputtering. Surf. Coatings Technol..

[13] Depla D., Buyle G., Haemers J., De Gryse R. (2006). Discharge voltage measurements during magnetron sputtering. Surf. Coatings Technol..

[14] De Graaf A., van Deelen J., Poodt P., van Mol T., Spee K., Grob F., Kuypers A. (2010). Development of atmospheric pressure CVD processes for high-quality transparent conductive oxides. Energy Procedia.

[15] Rho W.Y., Lee K.H., Han S.H., Kim H.Y., Jun B.H. (2019). Au-embedded and carbon-doped freestanding TiO_2_ nanotube arrays in dye-sensitized solar cells for better energy conversion efficiency. Micromachines.

[16] Kim K.-C., Lee D.-H., Maeng S. (2012). Synthesis of novel pure SnO nanostructures by thermal evaporation. Mater. Lett..

[17] Shimizu M., Usui H., Sakaguchi H. (2014). Electrochemical Na-insertion/extraction properties of SnO thick-film electrodes prepared by gas-depositionitle of the article. J. Power Sources.

[18] Tariq Z., Butt F.K., Rehman S.U., Haq B.U., Aleem F., Li C. (2019). First-principles study of electronic and optical properties of sulfur doped tin monoxide: A potential applicant for optoelectronic devices. Ceram. Int..

[19] Chu X., Zhu X., Dong Y., Zhang W., Bai L. (2015). Formaldehyde sensing properties of SnO–graphene composites prepared via hydrothermal method. J. Mater. Sci. Technol..

[20] Kumar R., Kushwaha N., Mittal J. (2017). Superior, rapid and reversible sensing activity of graphene-SnO hybrid film for low concentration of ammonia at room temperature. Sensors Actuators B Chem..

[21] Jeong H.-S., Park M.-J., Kwon S.-H., Joo H.-J., Song S.-H., Kwon H.-I. (2018). Low temperature NO_2_ sensing properties of RF-sputtered SnO-SnO_2_ heterojunction thin-film with p-type semiconducting behavior. Ceram. Int..

[22] Li N., Fan Y., Shi Y., Xiang Q., Wang X., Xu J. (2019). A low temperature formaldehyde gas sensor based on hierarchical SnO/SnO_2_ nano-flowers assembled from ultrathin nanosheets: Synthesis, sensing performance and mechanism. Sensors Actuators B Chem..

[23] Schiavi P.G., Altimari P., Rubino A., Pagnanelli F. (2018). Electrodeposition of cobalt nanowires into alumina templates generated by one-step anodization. Electrochim. Acta.

[24] Schiavi P.G., Farina L., Zanoni R., Altimari P., Cojocariu I., Rubino A., Navarra M.A., Panero S., Pagnanelli F. (2019). Electrochemical synthesis of nanowire anodes from spent lithium ion batteries. Electrochim. Acta.

[25] Prasad K.R., Miura N. (2004). Electrochemical synthesis and characterization of nanostructured tin oxide for electrochemical redox supercapacitors. Electrochem. Commun..

[26] Shi L., Xu Y., Li Q. (2010). Controlled fabrication of SnO_2_ arrays of well-aligned nanotubes and nanowires. Nanoscale.

[27] Yadava Y., Denicoló G., Arias A., Roman L., Hümmelgen I. (1997). Preparation and characterization of transparent conducting tin oxide thin film electrodes by chemical vapour deposition from reactive thermal evaporation of SnCl_2_. Mater. Chem. Phys..

[28] Saikia P.K., Borthakur A., Saikia P.K. (2011). Structural, optical and electrical properties of tin oxide thin film deposited by APCVD method. Indian J. Phys..

[29] Yang T., Zhao J., Li X., Gao X., Xue C., Wu Y., Tai R. (2012). Preparation and characterization of p-type transparent conducting SnO thin films. Mater. Lett..

[30] Caraveo-Frescas J.A., Alshareef H.N. (2013). Transparent p-type SnO nanowires with unprecedented hole mobility among oxide semiconductors. Appl. Phys. Lett..

[31] Shi J.-B., Wu P.-F., Lin H.-S., Lin Y.-T., Lee H.-W., Kao C.-T., Liao W.-H., Young S.-L. (2014). Synthesis and characterization of single-crystalline zinc tin oxide nanowires. Nanoscale Res. Lett..

[32] Shi J.-B., Wu P.-F., Lin Y.-T., Kao C.-T., Chen C.-J., Cheng F.-C., Liu H.-H., Chen Y.-C., Lin H.-S., Lee H.-W. (2015). Synthesis and optical properties of single-crystalline indium zinc oxide (IZO) nanowires via co-deposition and oxidation methods. Vacuum.

[33] Shi J.-B., Wu P.-F., Lin Y.-T., Chen C.-J., Kao C.-T., Lin H.-S., Lee H.-W., Liu H.-H., Lee M.-W., Chen C.-Y. (2015). Preparation and characterization of single crystalline In-Sn oxide nanowires. Vacuum.

[34] Yu H., Yang T., Wang Z., Li Z., Zhao Q., Zhang M. (2018). ρ-N heterostructural sensor with SnO-SnO_2_ for fast NO_2_ sensing response properties at room temperature. Sensors Actuators B Chem..

[35] Willa C., Yuan J., Niederberger M., Koziej D. (2015). When Nanoparticles meet Poly(Ionic Liquid)s: Chemoresistive CO_2_ sensing at room temperature. Adv. Funct. Mater..

[36] Srinives S., Sarkar T., Hernández R., Mulchandani A. (2015). A miniature chemiresistor sensor for carbon dioxide. Anal. Chim. Acta.

[37] Hafiz S.M., Ritikos R., Whitcher T.J., Razib N.M., Bien D.C.S., Chanlek N., Nakajima H., Saisopa T., Songsiriritthigul P., Huang N.M. (2004). A practical carbon dioxide gas sensor using room-temperature hydrogen plasma reduced graphene oxide. Adv. Mater..

[38] Star A., Han T.-R., Joshi V., Gabiel J.-C.P., Grüner G. (2015). Nanoelectronic carbon dioxide sensors. Vacuum.

